# A Polymorphism at the 3′-UTR Region of the Aromatase Gene Is Associated with the Efficacy of the Aromatase Inhibitor, Anastrozole, in Metastatic Breast Carcinoma

**DOI:** 10.3390/ijms140918973

**Published:** 2013-09-13

**Authors:** Lei Liu, Yu-Xian Bai, Jian-Hua Zhou, Xiu-Wei Sun, Hong Sui, Wen-Jie Zhang, Heng-Heng Yuan, Rui Xie, Xiao-Li Wei, Ting-Ting Zhang, Peng Huang, Yan-Jing Li, Jing-Xuan Wang, Shu Zhao, Qing-Yuan Zhang

**Affiliations:** Department of Internal Medicine, The Affiliated Tumor Hospital, Harbin Medical University, Haping Road 150 of Nangang District, Harbin 150081, Heilongjiang, China; E-Mails: 15331881117@yeah.net (L.L.); bai_yuxian@yeah.net (Y.-X.B.); zhoujianha_5@yeah.net (J.-H.Z.); sun_xiuwei@yeah.net (X.-W.S.); sui_hong@yeah.net (H.S.); zhang_wenjie@yeah.net (W.-J.Z.); yuanhengheng@yeah.net (H.-H.Z.); xie_rui@yeah.net (R.X.); wei_xiaoli@yeah.net (X.-L.W.); zhang_tingting@yeah.net (T.-T.Z.); huang_peng@yeah.net (P.H.); li_yanjing@yeah.net (Y.-J.L.); wang_jingxuan@yeah.net (J.-X.W.); zhao_shu@yeah.net (S.Z.)

**Keywords:** metastatic breast carcinoma, aromatase gene, anastrozole, prognosis, polymorphism

## Abstract

Estrogen-related genes and the fat mass and obesity-associated (*FTO*) gene play a critical role in estrogen metabolism, and those polymorphisms are associated with a poor prognosis in breast cancer. However, little is known about the association between these polymorphisms and the efficacy of anastrozole. The aim was to investigate the impact of the genetic polymorphisms, *CYP19A1*, *17-β-HSD-1* and *FTO*, on the response to anastrozole in metastatic breast carcinoma (MBC) and to evaluate the impact of those polymorphisms on various clinicopathologic features. Two-hundred seventy-two women with hormone receptor-positive MBC treated with anastrozole were identified retrospectively. DNA was extracted from peripheral blood and genotyped for five variants in three candidate genes. Time to progression was improved in patients carrying the variant alleles of rs4646 when compared to patients with the wild-type allele (16.40 months *versus* 13.52 months; *p* = 0.049). The rs4646 variant alleles were significantly associated with longer overall survival (37.3 months *versus* 31.6 months; *p* = 0.007). This relationship was not observed with the rs10046, rs2830, rs9926298 and rs9939609 polymorphisms. The findings of this study indicate that rs4646 polymorphism in the *CYP19A1* gene may serve as a prognostic maker of the response to anastrozole in patients with MBC who are treated with anastrozole.

## 1. Introduction

Breast cancer is one of the most prevalent malignancies in women worldwide. In 2012, 226,870 women were diagnosed with breast cancer in the United States, and nearly three million people are estimated to be living in the United States with a history of invasive breast cancer [1]. In China, breast cancer was the sond cause of incidence rates among all the cancers, with both increasing incidence and mortality [2]. Currently, aromatase inhibitors (AIs) are used in the treatment of metastatic breast cancer [3,4] and in the adjuvant setting [5–7]. Patients using AIs are proven to achieve significantly longer overall and disease-free survival than those who receive tamoxifen alone [6–11]. Two thirds of breast cancer cases express estrogen (ER) and/or progesterone receptors (PgR) [12–14]; positivity of estrogen or progesterone receptors in breast cancer has been related to the efficacy of tamoxifen, as well as AIs. To date, only the presence and intensity of hormonal receptors (ER/PgR) are useful tools as predictive markers in clinical practice. However, the clinical relevance of hormone receptors when using AIs is moderate, because only 30% of the patients exhibit an objective clinical response [15,16], and therefore, discriminating responders from non-responders may potentially be challenging. Predictive biomarkers to the response of treatment with aromatase inhibitors are an area of active investigation.

Two genes are considered to control the conversion rates of androgens (testosterone or androstenedione) into their parallel estrogens (estradiol or estrone). These are the aromatase gene (*CYP19A1*) and the type 1 17-β hydroxysteroid dehydrogenase (*17-β-HSD-1*) gene encoding the bidirectional enzyme that converts estrone (E1) to estradiol (E2) [17,18]. Previous studies have reported that *CYP19A1* polymorphisms are related to breast cancer risk in healthy women, and several *CYP19A1* gene variants are associated with a lower cancer risk [19–22]. Moreover, polymorphisms in the aromatase *CYP19A1* gene have been shown to alter aromatase activity in postmenopausal women [23,24]. No study of *CYP19A1* polymorphisms has addressed the possible relationships between these variants and the efficacy of anastrozole.

*17-β-HSD-1* is essential for the production of active E2 from E1 [25]. *17-β-HSD-1* is an independent prognostic factor in breast cancer in both pre- and post-menopausal patients [26]. Polymorphisms in the *17-β-HSD-1* gene have been shown to alter E2 levels in postmenopausal women [27]. There was almost a 34%–46% difference in postmenopausal E2 level according to obese *vs.* nonobese and *17-β-HSD-1* polymorphisms. However, no evidence between *17-β-HSD-1* polymorphisms and the therapeutic efficacy of anastrozole in MBC has yet been established.

In postmenopausal women, the primary source of estrogen is adipose tissue. Obesity may contribute to the risk of developing breast cancer and to a poorer prognosis [28]. The fat mass and obesity-associated (*FTO*) gene is associated with obesity in the general population [29], and there is evidence that single nucleotide polymorphisms (SNPs) located in intron 1 of *FTO* are associated with increased breast cancer risk [30]. Polymorphisms in estrogen-related genes and the *FTO* gene may predict better response to aromatase inhibitors and may be a prognostic factor for improved survival in metastatic breast carcinoma. In this retrospective study, we studied the impact of polymorphisms in *CYP19A1*, *17-β-HSD-1* and *FTO* on clinical outcomes in patients with hormone receptor-positive MBC who were treated with anastrozole. In addition, we hypothesized different response rates and survival in *CYP19A1* variants (rs10046 and rs4646), *17-β-HSD-1* variants (rs2830) or *FTO* variants (rs992628 and rs993960).

## 2. Results and Discussion

### 2.1. Results

#### 2.1.1. Patient, Treatment and Tumor Characteristics

A total of 272 women were included, with a median age of 63 years old (ranging from 36 to 85 years). The median Eastern Cooperative Oncology Group performance status was zero (range: 0–2), and all patients received anastrozole 1 mg orally once daily. The median number of metastatic locations for all patients was three (range: 1–5). Only 16 of 272 patients were obese (body mass index (BMI) ≥ 30.0 kg/m^2^). The mean baseline BMI was 24.0 kg/m^2^ (standard deviation: 3.6). All patients were evaluated for HER2, and 96 (35%) were found to have *HER2* gene amplification by filter *in situ* hybridization (FISH) and/or 3+ HER2 protein overexpression, and 36 (38%) patients received trastuzumab treatment as adjuvant therapy or treatment for metastatic cancer. Adverse events were recorded in 200 cases, and there were none recorded in 72 cases. None of the patients in this study abandoned anastrozole therapy due to side effects (Table 1).

#### 2.1.2. Allele Frequencies

The observed percentage of cases with SNP variations of rs10046 was 80.88%, of rs4646, 50%, and of rs9926298, 17.65%. The variation frequencies were 16.18% for rs9939609 and 80.88% for the rs2830 variations. Polymorphism references, genotypes, gene and chromosome locations and the frequency distribution of observed genotypes in the series are shown in Table 2. In addition, values for the Hardy-Weinberg (HW) equilibrium were estimated for each polymorphism and are also listed in Table 2. Five SNPs were in HW equilibrium.

#### 2.1.3. Clinical, Pathological, Genotypic Parameters and the Clinical Response to Anastrozole

Among the 272 metastatic breast cancer patients who received anastrozole therapy, a total of 56 patients showed partial responses (PR), 108 women demonstrated stable disease (SD) for more than six months and 32 patients achieved complete responses (CR) and were classified to have clinical benefit. We observed no correlation of SNPs with age, performance status, the number of metastatic sites, estrogen or progesterone receptor status or HER2 status (Table 3). Previous studies have demonstrated that rs4646 is associated with improved treatment efficacy in patients with hormone receptor-positive metastatic breast cancer treated with letrozole [31]. In our study, 32 patients best response to anastrozole was a complete response, 24 (75%) had a variant rs4646 SNP, whereas 60 of 76 (78.9%) of cases with progressive disease had a wild-type (GG) rs4646 SNP. The objective response categories were significantly different when segregated with respect to rs4646 status (Table 4); significance was maintained after applying the Bonferroni correction. This relationship was not observed with the rs10046, rs2830, rs9926298 and rs9939609 polymorphisms (data not shown). In addition, we analyzed the musculoskeletal adverse events of anastrozole in relation to the SNPs. The proportion of patients with treatment-related musculoskeletal adverse events was not different when stratified by *rs4646* status (Table 4), rs10046 status, rs2830 status, rs9926298 status and rs9939609 status (data not shown).

We further analyzed the association between different HER2/neu status and SNPs. Patients with a CC genotype of rs10046 had a HER2-positive tumor in 18 (18/52, 34.6%) of cases, and those with a CT or TT genotype in 52 (52/148, 27.0%) and 26 (26/72, 27.8%) of cases, respectively (*p* > 0.05). Similarly, HER2 status was not associated with the SNP rs4646 genotype. Patients with a GG genotype of rs4646 had a HER2-positive tumor in 52 (52/136, 38.2%) of cases, and those with a GT or TT genotype, in six (6/16, 37.5%) and 38 (38/120, 31.7%) of cases, respectively (*p* > 0.05).

#### 2.1.4. SNPs, TTP and OS

**Figure 1:**
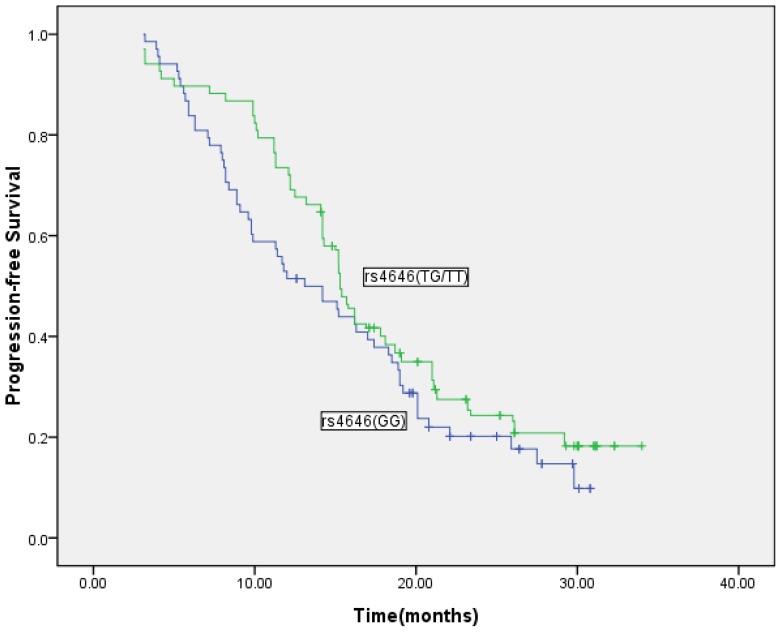
Time to progression segregated on the absence or presence of the rs4646 SNP variant (*n* = 262; normal: GG; variant: GT or TT). Log-rank, *p* = 0.049.

The median follow-up time was 72 months (ranging from 48 to 102 months). The median duration of anastrozole treatment was 18.1 months (range: 3.3–34 months), and the median time to progression (TTP) was 15.50 months. There were 208 deaths. Overall, the median survival time (MST) was 36.05 months. In our study, TTP in the 262 evaluable patients was significantly prolonged in the cases with the rs4646 SNP variant of *CYP19A1*, when compared with those showing the wild-type (WT) form of the gene (16.4 months *versus* 13.52 months; *p* = 0.049; Figure 1). The hazard risk was 0.50 (95% confidence interval, 0.27–0.93). This relationship was not observed for the rs10046 variant (14.93 months *versus* 16.89 months; *p* = 0.94), for the rs2830 variant (15.88 months *versus* 16.69 months; *p* = 0.095), for the rs9939609 variant of *FTO* (15.91 months *versus* 14.96 months; *p* = 0.089) or for the rs9926298 variant (16.16 months *versus* 15.33 months; *p* = 0.10). Multivariate analyses of TTP were done with rs4646 and known predictors of response, such as hormonal receptor subtypes, HER2 status, history of treatment with trastuzumab, number of metastatic locations, BMI levels and performance status. The statistical significance of rs4646 was <0.05 in all cases.

**Figure 2:**
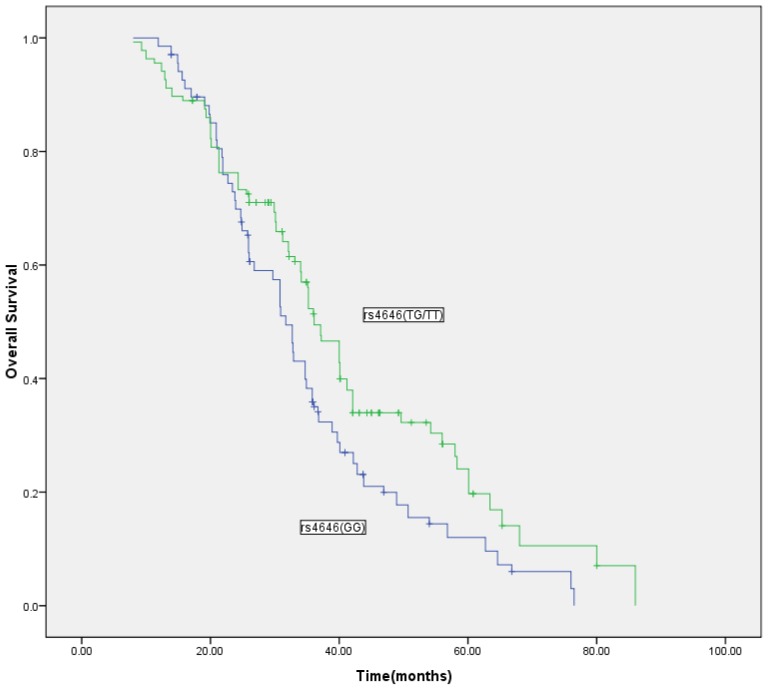
Overall survival segregated on the absence or presence of the rs4646 SNP variant (*n* = 262; normal: GG; variant: GT or TT). Log-rank, *p* = 0.007.

The rs4646 variant was associated with increased overall survival in our study. Individuals with the variant genotype (GT or TT) had an MST of 37.3 months, whereas those with the wild-type genotype (GG) had an MST of 31.6 months (log-rank test, *p* = 0.007; Figure 2). In the Cox proportional hazards model, after adjusting for the number of metastatic locations, performance status, previous treatment, hormonal receptor subtypes, HER2 status, history of treatment with trastuzumab and family history, we found that the hazard ratio (HR) was significantly higher for individuals with the wild-type genotype (GG) (HR *=* 2.37 95% confidence interval (CI) 1.20, 4.65; *p* = 0.001). None of the other genotypes of rs10046, rs2830, rs9939609 or rs9926298 were associated with the overall survival (OS) (data not shown).

### 2.2. Discussion

In this retrospective study, we evaluated the role of two estrogen-related gene polymorphisms and the *FTO* gene polymorphisms in the clinical outcome of anastrozole-treated MBC. To the best of our knowledge, this is the first study that investigates the association between estrogen-related genes and *FTO* polymorphisms and the clinical outcome of anastrozole therapy. We found that the rs4646 variant of the *CYP19A1* gene is associated with greater efficacy of anastrozole administered in postmenopausal women with MBC and retained statistical significance after Bonferroni correction.

In the case of MBC, population-based studies of common *CYP19A1* polymorphisms have generated inconsistent results with regard to their possible association with the efficacy of AIs or survival. It has been reported that in postmenopausal MBC women treated with letrozole, TTP was significantly prolonged in those with the rare T allele of rs4646 compared with homozygotes for the wild-type variant (GG) [31]. On the other hand, the same variants (GT and TT) were associated with a poorer benefit from letrozole (shorter progression-free survival), evaluated in a neoadjuvant setting [32]. Ghimenti *et al.* [33] evaluated the presence of rs6493497 and rs7176005 polymorphisms (mapping in the 5′ flanking region of the *CYP19A1* gene coding for the aromatase protein) in a cohort of 37 patients with postmenopausal breast cancer who received three-month neoadjuvant treatment with anastrozole. No association was found with response to anastrozole neoadjuvant treatment, with aromatase mRNA basal expression level or expression difference after therapy. In our study, half (50%) of patients had the variant form of the gene, and patients with the rs4646 SNP variant had a longer TTP and survival; this finding can be of considerable clinical relevance. The difference in our results, as compared to those of other investigators, may partly be explained by the different patient population of our study, which consisted of metastatic breast cancer patients previously treated with tamoxifen. Previous studies demonstrate that tamoxifen resistance in breast cancer may activate PI3K/AKT signaling, including *HER2*, type 1 insulin-like growth factor receptor (*IGF1R*) and activated mutant AKT1 [34,35]. However*,* polymorphisms in promoter regions of the aromatase gene may affect the levels of gene expression and reverse resistance to endocrine therapy. Moreover, there was a significantly greater suppression of Ki67 in breast cancer patients previously treated with tamoxifen therapy [36].

The present study demonstrates that an association between clinical outcome of anastrozole therapy and genetic variant are biologically plausible for the following reasons. First, for metastatic breast cancer, most SNPs are “silent” and do not alter the function or the expression of a gene [37]; the *CYP19A1* SNP that we have described seems to be active. This could be related to an advantage induced in the CYP19A1 protein structure that makes it more active. sond, either altered transcription and translation of the rs4646 genotype or another noncoding region polymorphism that is in linkage disequilibrium with the 3′-UTR variation are the most probable explanations for an effect of these 3′-UTR *CYP19A1* variations. The polymorphisms in *CYP19A1* 3′-UTR could reduce levels of circulating sex hormone [23,38] and alter protein activation, perhaps changing the biological activities, thus inducing the greater efficacy of AI.

Breast cancer population-based studies of common *CYP19A1* polymorphisms have generated inconsistent results with regard to their possible association with sex hormone levels or HER2 status [23,39–41]. In a previous study, the aromatase genotype reached significance even in a logistic regression model with grading, estrogen receptor status, progesterone receptor status and rs10046 and rs4646 status as independent variables and HER2 status as the dependent variable [40]. On the assumption that the variant genotype of rs10046 and rs4646 results in decreased aromatase activity, this could be an explanation for the lower percentage of HER2-positive tumors in patients with variant genotypes of the 3′-UTR polymorphisms in *CYP19A1*. Neoadjuvant therapy with aromatase inhibitors has been shown to result in a change in HER2 status [42]. The proportion of HER2 over-expression (immunohistochemistry 2+ and 3+) changed from 44.4% to 13.9% with aromatase inhibitor therapy. The proportion of patients amplifying the *HER2* gene also decreased during anti-aromatase therapy. In the current study, we also investigated the association of the rs4646 variant allele and the rs10046 variant allele with tumor HER2 status. No association between the HER2 status, hormonal status, TTP or OS of either rs10046 or rs4646 was observed in our series. This study needs further confirmation through functional analysis of tumor characteristics at the mRNA and protein levels.

Obesity on recurrence risk in women under AI treatment may not be due to incomplete aromatase inhibition [43]. The result also suggested that obese women under AI treatment have estradiol levels similar to lean women under AI treatment. This proposes that standard AI dosage is sufficient to inhibit aromatase in obese women. Two previous studies in advanced breast cancer did not find an association with BMI dependency and the efficacy of anastrozole treatment [44,45]. Our study showed similar results, in that anastrozole was equally efficient on all BMI levels in MBC. Additionally, no associations between the anastrozole treatment efficacy of either rs9926298 or rs9939609 were observed in our series. Of note, the low number of rs9926298 or rs9939609 variant alleles in this subgroup (17.65% and 16.18%) would hinder making such an association if present. Therefore, a much larger prospective study would be needed to effectively examine our conclusion.

Whether anastrozole therapy has modified clinical outcomes when examined in association with the other genotypes is unknown at present. In our cohort, the *CYP19A1* rs10046 variant allele gene frequency was 80.88% (heterozygous variant and homozygous variant frequency was 54.41% and 26.47%, respectively), the *17-β-HSD-1* rs2830 variant allele gene frequency was 80.88% (heterozygous variant and homozygous variant frequency was 52.94% and 27.94%, respectively) and other SNP variant allele frequencies were less than 20%. There were no significant differences of these proportions when compared with different genotypes.

There are some limitations to the current study. First, this is a retrospective study of patients who had various treatments before anti-aromatase therapy. Therefore, only the preliminary effects of SNPs on differential responses to anastrozole were investigated. These results should be confirmed using a larger prospective study. sond, our study was limited to examining the anastrozole clinical response and the association with several known clinicopathological factors. More studies are required to evaluate these genetic variants with other AI correlative factors, such as estradiol and estrone levels. Finally, the number of SNPs tested was limited to five, because of the exploratory nature of the study. However, the advantages of our study were that all patients were recruited from the same area and were matched for age, ethnicity and residence, and the SNP distributions were in Hardy-Weinberg equilibrium. Moreover, the cases were pathologically confirmed, and a strict quality control protocol was followed for genotype detection. Additionally, we concentrated on overall survival, as it is the most objective clinical outcome.

## 3. Experimental Section

### 3.1. Study Population

The medical records of patients who had received at least four-week treatment with anastrozole at the Affiliated Tumor Hospital of Harbin Medical University for hormone-receptor-positive postmenopausal MBC patients, between January 2002, and March 2010, were retrospectively reviewed. It was also necessary for a blood sample from the patient to be available for biological marker evaluation. Detailed eligibility criteria for this study were: (a) they were estrogen receptor-positive and/or progesterone receptor-positive (>10%); (b) the adequacy of clinical data on the patient’s history, demographics, tumor characteristics, treatment details (drug dosages, schedule of administration, serious toxicities) and clinical outcome; (c) metastatic breast cancer previously treated with tamoxifen and proven objective tumor progression; and (d) they received anastrozole treatment for metastatic disease. Exclusion criteria were: anastrozole was used as neo-adjuvant therapy or adjuvant hormonal treatment; if other cancers were experienced previously. Written informed consent was obtained from all patients for the use of blood cells for the analysis of genetic polymorphisms in association with clinical findings, including response to anastrozole. This study was approved by the Institutional Ethics Committee of the Affiliated Tumor Hospital of Harbin Medical University.

We evaluated clinical data and blood samples from patients with MBC, and what was thought to be estrogen receptor-positive and/or progesterone receptor-positive disease by locally performed immunohistochemistry. Additionally, breast cancer tumors were classified as HER2-positive if they demonstrated HER2 gene amplification by the *in situ* hybridization method or were scored as 3+ by an immunohistochemistry method. Musculoskeletal adverse events cases were defined to have had at least one of the following six multiplicative joint effects: joint pain, muscle pain, bone pain, arthritis, diminished joint function or other musculoskeletal problems. Cases were required to either (1) have at least grade 3 toxicity, according to the National Cancer Institute’s (NCI’s) Common Terminology Criteria for Adverse Events v3.0 or (2) go off treatment for any grade of musculoskeletal adverse events within the first 2 years (*i.e.*, a musculoskeletal adverse event occurring after 2 years was not considered a case). Characteristics of these patients are shown in Table 1.

### 3.2. DNA Extraction and Genotyping

Genomic DNA was isolated from the peripheral blood of 272 MBC patients using standard methods (Axygen, Union City, CA, USA). Polymerase Chain Reaction (PCR) amplification of genomic DNA was performed, followed by direct DNA sequencing to identify potential SNPs in *CYP19A1* (rs10046 and rs4646), *17-β-HSD-1* (rs2830) and *FTO* (rs9926298 and rs9939609). Primer pairs based on the published gene sequences were designed as follows: primers for SNPs in *CYP19A1* (rs10046 and rs4646), F-5′-CCTTGCACCCAGATGAGAC-3′ and R-5′-CAGAGGCCAAGAGTTTGAGG-3′; primers for rs2830 in *17-β-HSD-1*, F-5′-GACCCACTCTGGAATGAGGA-3′ and R-5′-CACCT GCTTGTAAAGCCTCC-3′; and primers for SNPs in *FTO* (rs9926298 and rs9939609), F-5′-TCA AAACTGGCTCTTGAATGAA-3′ and R-5′-AGAAATGGAGTGGGAGAGCA-3-. PCR reactions were performed in a total volume of 25 μL containing genomic DNA (25 ng), 1 μL of forward and reverse primers (10 μmol/L), 12.5 μL of PCR Master Mix (Tiangen Biotech, Beijing, China) and H_2_O (8.5 μL). PCR cycling was performed with an initial denaturation at 94 °C for 3 min, followed by 30 cycles of denaturation at 94 °C for 30 s, annealing at 56 °C for 30 s and extension at 72°C for 30 s, with a final extension at 72 °C for 5 min. PCR products were purified using a QIAquick Gel Extraction kit (Qiagen, Dusseldorf, Germany). Direct sequencing of PCR products were performed with an AB3730 DNA Analyzer (Applied Biosystems, Foster City, CA, USA). The reaction mixture contained 1 μL of PCR products, 1.6 μL of forward and reverse primers (same as PCR primers), H_2_O (1.4 μL) and Bigdye (1 μL). The reaction mixture was denatured at 96 °C for 1 min, followed by 30 cycles of 96 °C for 10 s, 50 °C for 5 s and 60 °C for 4 min. The Bigdye-labeled PCR products were sequenced using a genetic analyzer, and SNPs were checked by comparison with the published *CYP19A1*, *17-β-HSD-1* and *FTO* sequences.

### 3.3. Statistical Analysis

Data on selected patient and tumor characteristics, previous and subsequent lines of treatment, disease progression events and survival were obtained from medical records. Response data were collected from imaging reports where available and serial clinical assessments. Data were entered into a central database, and follow-up information for all patients was updated in February, 2012. Patients were divided into two categories: clinical benefit patients were those with a complete response, partial response or stable disease for >6 months; and no benefit patients were those with progressive disease (PD) or stable disease for ≤6 months. All examined polymorphisms are presented as frequency, while associations with the efficacy of the anastrozole were examined using the chi-square test or Fisher’s exact test, where appropriate. Time to progression (TTP) was defined as the time from anastrozole treatment for metastatic disease to the date of documented disease progression. Overall survival (OS) was defined as the time from anastrozole treatment for metastatic disease until death from any cause and was censored at the last follow-up. Median follow-up time was computed among censored observations only. The association between overall survival and the genetic polymorphisms was estimated using the method of Kaplan and Meier and assessed using the log-rank test. For the multivariate analyses, Cox proportional hazards models were used. *p* < 0.05 was considered significant, including, in the initial step, clinical parameters, such as: age, performance status, receptor status (ER/PgR), body mass index (BMI) levels, family history, number of metastatic sites and HER2 status. Genotypic frequencies were tested for Hardy-Weinberg equilibrium using the chi-square test [46]. All statistical analyses were performed using the SPSS (version 16.0) software for Windows.

## 4. Conclusions

Our extensive analysis of five SNPs in estrogen-related genes and the *FTO* gene identified the rs4646 as a strong candidate for defining inter-individual differences in the response to anastrozole treatment of MBC. Our results indicate the significant association of the rs4646 polymorphism in the 3′-UTR of *CYP19A1* in the outcome of patients with MBC and, also, suggest the utility of this SNP as a prognostic factor in anastrozole-treated patients with MBC. Further studies based on larger patient series are necessary to validate the predictive value of variant rs4646 and to confirm these findings.

## Figures and Tables

**Table 1:** Patients’ characteristics.

Ethnicity	*n* (%)
Chinese	272 (100%)
Age 1st diagnosis	Years
Median (range)	54 (31 to 84)
Age at treatment	Years
Median (range)	63 (36 to 85)
Surgery status	*n* (%)
No surgery	16 (5.9%)
Surgery	256 (94.1%)
Histological diagnosis *	*n* (%)
IDC	194 (71.3%)
ILC	38 (14.0%)
Others	40 (14.7%)
ECOG performance status	*n* (%)
0	156 (57.4%)
1–2	116 (42.6%)
HER2 status	*n* (%)
Negative	176 (64.7%)
Positive	96 (35.3%)
ER status	*n* (%)
ER+/PR+	196 (72.1%)
ER+/PR−	52 (19.1%)
ER−/PR+	24 (8.8%)
Response to anastrozole **	*n* (%)
Non-responders	184 (72.1%)
Responders	88 (27.9%)
BMI level	*n* (%)
≤24	161(59.2%)
<24	111(40.8%)
No. of metastatic locations	
≤3	173 (63.6%)
>3	99 (36.4%)

* IDC, Infiltrating ductal carcinoma; ILC, Infiltrating lobular carcinoma; others (mucinous, tubular and medullar carcinomas); Abbreviations: ER, estrogen receptor; PR, progesterone receptor; ECOG, Eastern Cooperative Oncology Group; BMI, body mass index;

** Responders, partial response and complete response; non-responders, stable disease and disease progression.

**Table 2:** Characteristics of five SNPs in the three genes analyzed.

Gene	Reference sequence	db-SNP ID	Genotype	*n* (%)	p*	Gene location	Chromosome position
*CYP19A1*	NT_010194.17	rs10046	CC	52 (19.12)	0.438	3′-UTR	5102986
			TT	72 (26.47)			
			CT	148 (54.41)			
		rs4646	GG	136 (50.00)	0.209	3′-UTR	51502844
			TT	16 (5.88)			
			GT	120 (44.12)			
*17-β-HSD-1*	NT_010783.15	rs2830	GG	52 (19.12)	0.580	5′-UTR	40704563
			AA	76 (27.94)			
			AG	144 (52.94)			
*FTO*	NT_010498.15	rs9926298	GG	224 (82.35)	0.425	Intron 1	80254332
			AA	0 (0)			
			AG	48 (17.65)			
		rs9939609	TT	228 (83.82)	0.468	Intron 1	53820527
			AA	0 (0)			
			AT	44 (16.18)			

* *p* > 0.05 is consistent with the Hardy-Weinberg equilibrium.

**Table 3:** Correlations of rs4646 and clinical variables.

Variable		*CYP19* gene rs4646 (*n* = 136)		

	*n*	WT, *n* (%)	Variant, *n* (%)	*p*
*Histological diagnosis*				
IDC	194	98 (72.1)	96 (70.6)	1.000
ILC	38	20 (14.7)	18 (13.2)	
Others	40	18 (13.2)	22 (16.2)	
*ECOG performance status*				
0	156	76 (55.9)	80 (58.8)	1.000
1–2	116	60 (44.1)	56 (41.2)	
*BMI level*				
≤24	161	75 (55.1)	86 (63.2)	1.000
>24	111	61 (44.9)	50 (36.8)	
*No. of metastatic locations*				
≤3	173	95 (69.9)	78 (57.4)	1.000
>3	99	41 (30.1)	58 (42.6)	
*HER2 status*				
Negative	176	84 (61.8)	92 (67.6)	1.000
Positive	96	52 (38.2)	44 (32.4)	
*Trastuzumab therapy history*				
Yes	36	16 (30.8)	20 (45.5)	1.000
No	60	36 (69.2)	24 (54.5)	
*Family history*				
No	180	80 (58.8)	100 (73.5)	1.000
Yes	92	56 (41.2)	36 (26.5)	
*ER status*				
ER+/PR+	196	96 (70.6)	100 (73.5)	1.000
ER+/PR−	52	24 (17.6)	28 (20.6)	
ER−/PR+	24	16 (11.8)	8 (5.9)	

Note: WT, wild-type (GG); variant, heterozygous or homozygous for the SNP rs4646 (GT or TT).

**Table 4:** Correlation between response to treatment and adverse effects segregated with respect to SNP rs4646.

Response and adverse events		*CYP19* gene rs4646 (*n* = 136)		

	*n*	WT, *n* (%)	Variant, *n* (%)	*p*
Best response				
Complete response	32	8 (5.9)	24 (17.6)	<0.005
Partial response	56	16 (11.8)	40 (29.4)	
Stable disease	108	52 (38.2)	56 (41.2)	
Disease progression	76	60 (44.1)	16 (11.8)	
Clinical benefit				
Benefit*	196	76 (55.9)	120 (88.2)	
No benefit	76	60 (44.1)	16 (11.8)	<0.005
Adverse effects				
No	72	40 (29.4)	32 (23.5)	0.894
Yes	200	96 (70.6)	104 (76.5)	

NOTE: WT, wild-type (GG); variant, heterozygous or homozygous for the SNP rs4646 (GT or TT);

* Clinical benefit = complete response + partial response + stable disease > 6 m.

## References

[1] Siegel R., de Santis C., Virgo K., Stein K., Mariotto A., Smith T., Cooper D., Gansler T., Lerro C., Fedewa S. (2012). Cancer treatment and survivorship statistics. CA Cancer J. Clin.

[2] Zhang M.L., Huang Z.Z., Zheng Y. (2012). Estimates and prediction on incidence, mortality and prevalence of breast cancer in China, 2008 (in Chinese). Zhonghua Liu Xing Bing Xue Za Zhi.

[3] Mouridsen H., Gershanovich M. (2003). The role of aromatase inhibitors in the treatment of metastatic breast cancer. Semin. Oncol.

[4] Campos S.M., Winer E.P. (2003). Hormonal therapy in postmenopausal women with breast cancer. Oncology.

[5] Howell A., Cuzick J., Baum M., Buzdar A., Dowsett M., Forbes J.F., Hoctin-Boes G., Houghton J., Locker G.Y., Tobias J.S. (2005). Results of the ATAC (Arimidex, Tamoxifen, Alone or in Combination) trial after completion of 5 years’ adjuvant treatment for breast cancer. Lancet.

[6] Goss P.E., Ingle J.N., Martino S., Robert N.J., Muss H.B., Piccart M.J., Castiglione M., Tu D., Shepherd L.E., Pritchard K.I. (2003). A randomized trial of letrozole in postmenopausal women after five years of tamoxifen therapy for early-stage breast cancer. N. Engl. J. Med.

[7] Coombes R.C., Hall E., Gibson L.J., Paridaens R., Jassem J., Delozier T., Jones S.E., Alvarez I., Bertelli G., Ortmann O. (2004). A randomized trial of exemestane after two to three years of tamoxifen therapy in postmenopausal women with primary breast cancer. N. Engl. J. Med.

[8] Baum M., Budzar A.U., Cuzick J., Forbes J., Houghton J.H., Klijn J.G., Sahmoud T., Group A.T. (2002). Anastrozole alone or in combination with tamoxifen *versus* tamoxifen alone for adjuvant treatment of postmenopausal women with early breast cancer: First results of the ATAC randomised trial. Lancet.

[9] Baum M., Buzdar A., Cuzick J., Forbes J., Houghton J., Howell A., Sahmoud T., Group A.T. (2003). Anastrozole alone or in combination with tamoxifen *versus* tamoxifen alone for adjuvant treatment of postmenopausal women with early-stage breast cancer: Results of the ATAC (Arimidex, Tamoxifen Alone or in Combination) trial efficacy and safety update analyses. Cancer.

[10] Jakesz R., Jonat W., Gnant M., Mittlboeck M., Greil R., Tausch C., Hilfrich J., Kwasny W., Menzel C., Samonigg H. (2005). Switching of postmenopausal women with endocrine-responsive early breast cancer to anastrozole after 2 years’ adjuvant tamoxifen: Combined results of ABCSG trial 8 and ARNO 95 trial. Lancet.

[11] Goss P.E., Ingle J.N., Martino S., Robert N.J., Muss H.B., Piccart M.J., Castiglione M., Tu D., Shepherd L.E., Pritchard K.I. (2005). Randomized trial of letrozole following tamoxifen as extended adjuvant therapy in receptor-positive breast cancer: updated findings from NCIC CTG MA.17. J. Natl. Cancer Inst.

[12] Bulun S.E., Lin Z., Imir G., Amin S., Demura M., Yilmaz B., Martin R., Utsunomiya H., Thung S., Gurates B. (2005). Regulation of aromatase expression in estrogen-responsive breast and uterine disease: From bench to treatment. Pharmacol. Rev.

[13] Toniolo P.G., Levitz M., Zeleniuch-Jacquotte A., Banerjee S., Koenig K.L., Shore R.E., Strax P., Pasternack B.S. (1995). A prospective study of endogenous estrogens and breast cancer in postmenopausal women. J. Natl. Cancer Inst.

[14] Paridaens R., Thomas J., Wildiers J., Vermeiren P., Lobelle J.P., di Salle E., Ornati G., Zurlo M.G., Polli A., Lanzalone S. (1998). Safety, activity and estrogen inhibition by exemestane in postmenopausal women with advanced breast cancer: A phase I study. Anticancer Drugs.

[15] Mouridsen H., Gershanovich M., Sun Y., Perez-Carrion R., Boni C., Monnier A., Apffelstaedt J., Smith R., Sleeboom H.P., Jaenicke F. (2003). Phase III study of letrozole *versus* tamoxifen as first-line therapy of advanced breast cancer in postmenopausal women: Analysis of survival and update of efficacy from the International Letrozole Breast Cancer Group. J. Clin. Oncol.

[16] Paridaens R., Dirix L., Lohrisch C., Beex L., Nooij M., Cameron D., Biganzoli L., Cufer T., Duchateau L., Hamilton A. (2003). Mature results of a randomized phase II multicenter study of exemestane *versus* tamoxifen as first-line hormone therapy for postmenopausal women with metastatic breast cancer. Ann. Oncol.

[17] Moeller G., Adamski J. (2009). Integrated view on 17beta-hydroxysteroid dehydrogenases. Mol. Cell Endocrinol.

[18] Khan N., Sharma K.K., Andersson S., Auchus R.J. (2004). Human 17beta-hydroxysteroid dehydrogenases types 1, 2, and 3 catalyze bi-directional equilibrium reactions, rather than unidirectional metabolism, in HEK-293 cells. Arch. Biochem. Biophys.

[19] Hirose K., Matsuo K., Toyama T., Iwata H., Hamajima N., Tajima K. (2004). The CYP19 gene codon 39 Trp/Arg polymorphism increases breast cancer risk in subsets of premenopausal Japanese. Cancer Epidemiol. Biomark. Prev.

[20] Miyoshi Y., Ando A., Hasegawa S., Ishitobi M., Yamamura J., Irahara N., Tanji Y., Taguchi T., Tamaki Y., Noguchi S. (2003). Association of genetic polymorphisms in CYP19 and CYP1A1 with the oestrogen receptor-positive breast cancer risk. Eur. J. Cancer.

[21] Haiman C.A., Stram D.O., Pike M.C., Kolonel L.N., Burtt N.P., Altshuler D., Hirschhorn J., Henderson B.E. (2003). A comprehensive haplotype analysis of CYP19 and breast cancer risk: The Multiethnic Cohort. Hum. Mol. Genet..

[22] Lee K.M., Abel J., Ko Y., Harth V., Park W.Y., Seo J.S., Yoo K.Y., Choi J.Y., Shin A., Ahn S.H. (2003). Genetic polymorphisms of cytochrome P450 19 and 1B1, alcohol use, and breast cancer risk in Korean women. Br. J. Cancer.

[23] Dunning A.M., Dowsett M., Healey C.S., Tee L., Luben R.N., Folkerd E., Novik K.L., Kelemen L., Ogata S., Pharoah P.D. (2004). Polymorphisms associated with circulating sex hormone levels in postmenopausal women. J. Natl. Cancer Inst.

[24] Mitrunen K., Hirvonen A. (2003). Molecular epidemiology of sporadic breast cancer. The role of polymorphic genes involved in oestrogen biosynthesis and metabolism. Mutat. Res.

[25] Puranen T., Poutanen M., Ghosh D., Vihko P., Vihko R. (1997). Characterization of structural and functional properties of human 17 beta-hydroxysteroid dehydrogenase type 1 using recombinant enzymes and site-directed mutagenesis. Mol. Endocrinol.

[26] Oduwole O.O., Li Y., Isomaa V.V., Mantyniemi A., Pulkka A.E., Soini Y., Vihko P.T. (2004). 17-β-hydroxysteroid dehydrogenase type 1 is an independent prognostic marker in breast cancer. Cancer Res.

[27] Sowers M.R., Randolph J.F., Zheng H., Jannausch M., McConnell D., Kardia S.R., Crandall C.J., Nan B. (2011). Genetic polymorphisms and obesity influence estradiol decline during the menopause. Clin. Endocrinol.

[28] Elme A., Utriainen M., Kellokumpu-Lehtinen P., Palva T., Luoto R., Nikander R., Huovinen R., Kautiainen H., Jarvenpaa S., Penttinen H.M. (2013). Obesity and physical inactivity are related to impaired physical health of breast cancer survivors. Anticancer Res.

[29] Gerken T., Girard C.A., Tung Y.C., Webby C.J., Saudek V., Hewitson K.S., Yeo G.S., McDonough M.A., Cunliffe S., McNeill L.A. (2007). The obesity-associated FTO gene encodes a 2-oxoglutarate-dependent nucleic acid demethylase. Science.

[30] Kaklamani V., Yi N., Sadim M., Siziopikou K., Zhang K., Xu Y., Tofilon S., Agarwal S., Pasche B., Mantzoros C. (2011). The role of the fat mass and obesity associated gene (FTO) in breast cancer risk. BMC Med. Genet.

[31] Colomer R., Monzo M., Tusquets I., Rifa J., Baena J.M., Barnadas A., Calvo L., Carabantes F., Crespo C., Munoz M. (2008). A single-nucleotide polymorphism in the aromatase gene is associated with the efficacy of the aromatase inhibitor letrozole in advanced breast carcinoma. Clin. Cancer Res..

[32] Garcia-Casado Z., Guerrero-Zotano A., Llombart-Cussac A., Calatrava A., Fernandez-Serra A., Ruiz-Simon A., Gavila J., Climent M.A., Almenar S., Cervera-Deval J. (2010). A polymorphism at the 3′-UTR region of the aromatase gene defines a subgroup of postmenopausal breast cancer patients with poor response to neoadjuvant letrozole. BMC Cancer.

[33] Ghimenti C., Mello-Grand M., Grosso E., Scatolini M., Regolo L., Zambelli A., Chiorino G. (2013). Regulation of aromatase expression in breast cancer treated with anastrozole neoadjuvant therapy. Exp. Ther. Med..

[34] Fan M., Yan P.S., Hartman-Frey C., Chen L., Paik H., Oyer S.L., Salisbury J.D., Cheng A.S., Li L., Abbosh P.H. (2006). Diverse gene expression and DNA methylation profiles correlate with differential adaptation of breast cancer cells to the antiestrogens tamoxifen and fulvestrant. Cancer Res.

[35] Miller T.W., Hennessy B.T., Gonzalez-Angulo A.M., Fox E.M., Mills G.B., Chen H., Higham C., Garcia-Echeverria C., Shyr Y., Arteaga C.L. (2010). Hyperactivation of phosphatidylinositol-3 kinase promotes escape from hormone dependence in estrogen receptor-positive human breast cancer. J. Clin. Invest.

[36] Dowsett M., Ebbs S.R., Dixon J.M., Skene A., Griffith C., Boeddinghaus I., Salter J., Detre S., Hills M., Ashley S. (2005). Biomarker changes during neoadjuvant anastrozole, tamoxifen, or the combination: Influence of hormonal status and HER-2 in breast cancer—A study from the IMPACT trialists. J. Clin. Oncol.

[37] Erichsen H.C., Chanock S.J. (2004). SNPs in cancer research and treatment. Br. J. Cancer.

[38] Haiman C.A., Dossus L., Setiawan V.W., Stram D.O., Dunning A.M., Thomas G., Thun M.J., Albanes D., Altshuler D., Ardanaz E. (2007). Genetic variation at the CYP19A1 locus predicts circulating estrogen levels but not breast cancer risk in postmenopausal women. Cancer Res.

[39] Probst-Hensch N.M., Ingles S.A., Diep A.T., Haile R.W., Stanczyk F.Z., Kolonel L.N., Henderson B.E. (1999). Aromatase and breast cancer susceptibility. Endocr. Relat. Cancer.

[40] Fasching P.A., Loehberg C.R., Strissel P.L., Lux M.P., Bani M.R., Schrauder M., Geiler S., Ringleff K., Oeser S., Weihbrecht S. (2008). Single nucleotide polymorphisms of the aromatase gene (CYP19A1), HER2/neu status, and prognosis in breast cancer patients. Breast Cancer Res. Treat.

[41] Long J.R., Kataoka N., Shu X.O., Wen W., Gao Y.T., Cai Q., Zheng W. (2006). Genetic polymorphisms of the CYP19A1 gene and breast cancer survival. Cancer Epidemiol. Biomark. Prev.

[42] Zhu L., Chow L.W., Loo W.T., Guan X.Y., Toi M. (2004). Her2/neu expression predicts the response to antiaromatase neoadjuvant therapy in primary breast cancer: Subgroup analysis from celecoxib antiaromatase neoadjuvant trial. Clin. Cancer Res.

[43] Diorio C., Lemieux J., Provencher L., Hogue J.C., Vachon E. (2012). Aromatase inhibitors in obese breast cancer patients are not associated with increased plasma estradiol levels. Breast Cancer Res. Treat.

[44] Bonneterre J., Thurlimann B., Robertson J.F., Krzakowski M., Mauriac L., Koralewski P., Vergote I., Webster A., Steinberg M., von Euler M. (2000). Anastrozole *versus* tamoxifen as first-line therapy for advanced breast cancer in 668 postmenopausal women: results of the Tamoxifen or Arimidex Randomized Group Efficacy and Tolerability study. J. Clin. Oncol.

[45] Bonneterre J., Buzdar A., Nabholtz J.M., Robertson J.F., Thurlimann B., von Euler M., Sahmoud T., Webster A., Steinberg M. (2001). Arimidex Writing Committee, *et al.* Anastrozole is superior to tamoxifen as first-line therapy in hormone receptor positive advanced breast carcinoma. Cancer.

[46] Stern C. (1943). The Hardy-Weinberg law. Science.

